# Risk factor analysis and risk prediction study of obesity in steelworkers: model development based on an occupational health examination cohort dataset

**DOI:** 10.1186/s12944-023-01994-x

**Published:** 2024-01-08

**Authors:** Zekun Zhao, Haipeng Lu, Rui Meng, Zhikang Si, Hui Wang, Xuelin Wang, Jiaqi Chen, Yizhan Zheng, Huan Wang, Jiaqi Hu, Ziqi Zhao, Hongmin Zhu, Jianhui Wu, Xiaoming Li, Ling Xue

**Affiliations:** School of Public Health, North China University of Science and Technology, No. 21 Bohai Avenue, Caofeidian New Town, Tangshan, 063210 China

**Keywords:** Obesity, Steelworkers, Risk factor analysis, Risk prediction, Random forest

## Abstract

**Background:**

Obesity is increasingly recognized as a grave public health concern globally. It is associated with prevalent diseases including coronary heart disease, fatty liver, type 2 diabetes, and dyslipidemia. Prior research has identified demographic, socioeconomic, lifestyle, and genetic factors as contributors to obesity. Nevertheless, the influence of occupational risk factors on obesity among workers remains under-explored. Investigating risk factors specific to steelworkers is crucial for early detection, prediction, and effective intervention, thereby safeguarding their health.

**Methods:**

This research utilized a cohort study examining health impacts on workers in an iron and steel company in Hebei Province, China. The study involved 5469 participants. By univariate analysis, multifactor analysis, and review of relevant literature, predictor variables were found. Three predictive models—XG Boost, Support Vector Machine (SVM), and Random Forest (RF)—were employed.

**Results:**

Univariate analysis and cox proportional hazard regression modeling identified age, gender, smoking and drinking habits, dietary score, physical activity, shift work, exposure to high temperatures, occupational stress, and carbon monoxide exposure as key factors in the development of obesity in steelworkers. Test results indicated accuracies of 0.819, 0.868, and 0.872 for XG Boost, SVM, and RF respectively. Precision rates were 0.571, 0.696, and 0.765, while recall rates were 0.333, 0.592, and 0.481. The models achieved AUCs of 0.849, 0.908, and 0.912, with Brier scores of 0.128, 0.105, and 0.104, log losses of 0.409, 0.349, and 0.345, and calibration-in-the-large of 0.058, 0.054, and 0.051, respectively. Among these, the Random Forest model demonstrated superior performance.

**Conclusions:**

The research indicates that obesity in steelworkers results from a combination of occupational and lifestyle factors. Of the models tested, the Random Forest model exhibited superior predictive ability, highlighting its significant practical application.

## Background

Obesity, a metabolic disorder, leads to various health and psychological issues [1]. The World Health Organization recognizes obesity as a major global public health challenge, impacting individual and societal health and escalating healthcare costs. Obesity risk factors are multifaceted, encompassing demographic and socioeconomic elements (age, gender, ethnicity, education, income, marital status, and residency) [2–5]; lifestyle factors (dietary status, smoking, alcohol consumption, and physical activity) [6–10]; and genetic influences [11, 12]. While some risk factors for obesity are immutable, others can be modified. Identifying modifiable risk factors is critical for developing effective prevention and intervention strategies to reduce obesity. For occupational groups, it is also essential to consider job-related exposure factors. Studies indicate that obesity prevalence in occupational groups significantly exceeds that in the general population [13]. This is particularly evident in the steel industry, characterized by diverse job roles, hazardous work conditions, a large workforce, limited health awareness, and unhealthy habits. A 2021 study of iron and steel workers in Beijing, Tianjin, and Hebei revealed an obesity rate of 63.16%, substantially higher than the 50.70% rate among Chinese adults [14], highlighting a major health risk for these workers.

Previous studies have shown that specific occupation-related factors in steel enterprises have a significant impact on obesity in steel workers [15, 16]. Steelworkers, frequently exposed to high temperatures, noise, and dust, and often engaged in shift work, face unique obesity risks compared to the general population. Given these findings, investigating occupational risk factors for obesity and devising protective strategies and measures is imperative. Early detection and lifestyle interventions for at-risk steelworkers can significantly reduce the incidence of obesity.

Recent advancements in medicine have seen the rapid evolution and widespread integration of machine learning (ML) technologies, particularly in diagnosing, prognosticating, and managing diseases. The use of ML to model epidemiological data is gaining prominence in published scientific literature. Compared to traditional methods, prior research suggests that ML techniques enhance the prediction of health outcomes [17]. While numerous studies have employed ML to forecast obesity prevalence [18–21], these models typically focus on disease risk within the general population and overlook specific characteristics of occupational groups. As a result, such models are not suitable for steelworkers. Consequently, there is a pressing need to develop a new obesity risk prediction model tailored to steelworkers, aiming to improve their health and quality of life. This study, using physical examination data of steelworkers from 2017 to 2022, aims to identify obesity risk factors specific to this group and determine the best obesity prediction model applicable to steelworkers.

## Methods

### Study subject

**Fig. 1:**
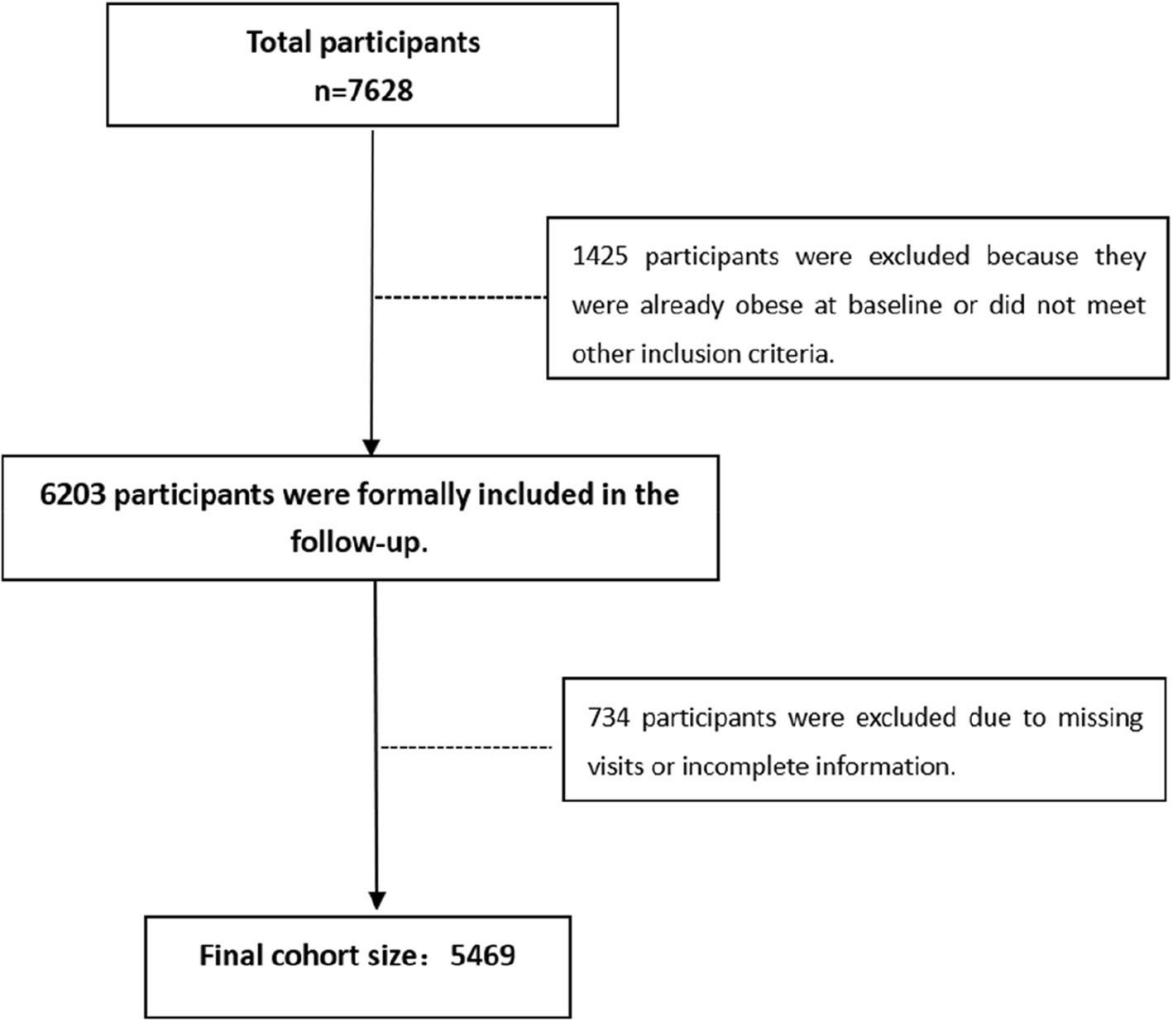
The process of study participant selection

This study draws from the “Cohort Study on Health Effects of Occupational Populations in Beijing-Tianjin-Hebei Region,” part of the National Key Research and Development Initiative. A baseline survey, conducted in January-September 2017, focused on workers in an iron and steel enterprise in Tangshan City (ISCO-08: 8122). Four follow-up data collections were completed in 2019, 2020, 2021, and 2022. Inclusion criteria were: age 18 to 60 years; regular employment status in the organization; a working tenure exceeding one year; and a non-obese status at baseline. Exclusion criteria included a working tenure of less than one year, being over 60 years of age, loss to follow-up, or incomplete survey information. All participants provided informed consent. The North China University of Technology Ethics Committee granted approval for the study on May 12, 2016, in accordance with the Declaration of Helsinki (approval number: 16,040). Figure 1 depicts the participant selection procedure.

### Information collection

The study employed a survey questionnaire and conducted one-on-one interviews with steelworkers, carried out by PhD and MSc students from North China University of Science and Technology. Licensed medical examiners followed standard testing procedures when doing physical assessments on these workers. Fasting blood samples were collected before 9:00 a.m. daily for laboratory analysis, utilizing a Myriad automatic biochemical analyzer (BS-800) for standard blood biochemical testing.

Data collection primarily included: (1) demographic information such as age, education level, marital status, and household income; (2) lifestyle habits like smoking, drinking, exercising, and diet; (3) physical and laboratory tests including blood biochemistry, height, and weight; and (4) occupational hazard exposure, covering aspects like shift work, service duration, dust, high temperatures, noise, and CO exposure.

### Obesity diagnostic criteria

Body mass index (BMI) was calculated by obtaining the height and weight of the survey respondents based on survey measurements. The criteria for defining obesity based on BMI differ slightly internationally, reflecting regional population characteristics. In 2002, China conducted an extensive epidemiological survey of over 240,000 adults across 21 provinces, including Taiwan [22, 23], and established its obesity criteria: a BMI of ≥ 28.0 kg/m^2^.

### Variable definition

#### Smoking

The three categories of smoking status among the participants were never smoked, former smoker, and current smoker, following the World Health Organization’s definitions [24]. ‘Current smoker’ denotes smoking for over six months at a minimum of one cigarette per day; ‘former smoker’ refers to those who had quit smoking for at least six months.

#### Drinking

Alcohol consumption was classified as never drinking, former drinker, or current drinker, as per guidelines from the Chinese Center for Disease Control and Prevention [25]. ‘Current drinker’ implies regular alcohol consumption for over six months, at least once per week; ‘former drinker’ denotes abstaining for at least six months.

#### Diet

The study assessed consumption of red meat, processed meats, sugary drinks, grains, vegetables, fruits, milk, nuts, and legumes, along with sodium intake. Dietary scores, based on the Dietary Approaches to Stop Hypertension (DASH) criteria [26], were assigned. Each food category was scored from 1 to 5 based on weekly intake frequency. The total dietary score ranged from 8 to 40. With a median DASH score of 25, this study divided dietary patterns into two categories: DASH < 25 and DASH ≥ 25.

#### Physical activity

This study assessed the physical activity of employees in the iron and steel industries using the International Physical Activity Questionnaire (IPAQ) [27]. The questionnaire covered daily work, transportation, lifestyle activities, exercise, recreation, sedentary time, and sleep duration. Each activity in the IPAQ was assigned a metabolic equivalent task (MET) value (Table 1). An individual’s weekly level of physical activity was calculated as MET × weekly frequency × daily duration. The intensity of various activities was summed to determine the total weekly physical activity level (MET-min/week). Based on intensity, frequency, and total weekly activity, physical activity levels were categorized as “low,” “medium,” or “high” (Table 2).

**Table 1:** The physical activity attributes and their MET assignments in the IPAQ long form

Type of physical activity	Physical Activity Program	Physical activity intensity	MET Assignment
Work Related	Walking	Walking	3.3
	Medium strength	Medium	4.0
	High Strength	High	8.0
Traffic and Travel Related	Walking	Walking	3.3
	Cycling	Medium	6.0
Home gardening related	Medium-intensity household chores	Medium	3.0
	Medium-intensity outdoor housework	Medium	4.0
	High-intensity outdoor housework	Medium	5.5
Leisure related	Walking	Walking	3.3
	Medium strength	Medium	4.0
	High Strength	High	8.0

**Table 2:** Individual physical activity level grouping criteria

Grouping	Standard
High	Meet any 1 of the following 2 criteria:
	Total high-intensity physical activity ≥ 3 d. Total weekly physical activity level ≥ 1500 MET-min/w;
	3 intensities of physical activity combined ≥ 7 d and a total weekly physical activity level of ≥ 3000 MET-min/w.
Medium	Meet any 1 of the following 3 criteria:
	At least 20 min of all types of vigorous physical activity per day for a total of ≥ 3 d;
	At least 30 min of all types of moderate light and/or walking activity per day for a total of ≥ 5 d;
	3 intensities of physical activity combined ≥ 5 d and a total weekly physical activity level of ≥ 600 MET-min/w.
Low	Meet any 1 of the following 2 criteria:
	No activity was reported;
	Some activities were reported but did not yet meet the above criteria for high and medium groupings.

#### High temperature

Following the national standard “Measurement of Physical Factors in the Workplace Part 7: High Temperature” [28], operations with a WBGT index ≥ 25 °C and a significant heat source are classified as high-temperature operations.

#### Noise

According to the national standard “Measurement of Physical Factors in the Workplace Part 8: Noise“ [29], operations are considered noisy if the equivalent sound level exposure is ≥ 80 dB(A) over 8 h per day or 40 h per week.

#### Dust exposure

Based on the national standard “Determination of dust in workplace air part 1: total dust concentration” [30]. Computation of cumulative personal dust exposure using the steel firm’s real daily testing data and an on-site total dust concentration test conducted by a qualified testing organization.

#### CO exposure

Following the national standard “Determination of Air Toxic Substances in Workplaces Inorganic Carbonaceous Compounds” [31], individual cumulative CO exposure was calculated based on on-site CO concentration assessments conducted by qualified testing companies and the daily actual test results from steel companies.

#### Occupational stress

A modified version of the work content questionnaire (JCQ) [32], was used to quantify occupational stress. It consisted of three dimensions: job demands (5 items), job autonomy (9 items), and social support (8 items). Each item was rated on a 1 to 4 scale, with the total score for each dimension reflecting job demands, autonomy, and social support levels. Occupational stress was assessed using the demand/control (D/C) ratio, calculated as follows:

1 $$\mathrm D/\mathrm{Cration}\frac{\mathrm{Job}\;\mathrm{requirement}\;\mathrm{factor}\;\mathrm{score}}{\mathrm{Degree}\;\mathrm{of}\;\mathrm{job}\;\mathrm{autonomy}\;\mathrm{factor}\;\mathrm{score}\;\mathrm x\;\mathrm C}$$

In this equation, C represents the ratio of job demand items to job autonomy items (5/9). If a D/C ratio ≤ 1 denotes the lack of occupational stress, a D/C ratio > 1 implies occupational stress.

Shift work: Shift work was categorized as never, former (previously but not currently shifted), and current.

Sleep quality: Sleep quality was evaluated using the Athens Insomnia Scale (AIS). This scale includes 8 items, each scored from 0 to 3, with the total score determining the AIS score. According to AIS criteria: AIS < 4 indicates no sleep disorder; 4 ≤ AIS ≤ 6 suggests suspected insomnia; AIS > 6 confirms insomnia.

### Sample size calculation

The model’s predictive accuracy was assessed based on the average outcome events. Reviewing literature revealed that the prevalence of obesity among steel company workers is 20.01% [14]. Placing a 0.05 margin of error (δ), a minimum of 248 study subjects was required, as demonstrated in Eq. (2),

2 $$\begin{array}{c}n=\left(\frac{1.96}\delta\right)^2\varphi\left(1-\varphi\right)\end{array}$$

The predictor variable p was roughly 20, and the mean absolute percentage error (MAPE) was set at 0.05 to indicate the least mean error for each predicted value [33]. Consequently, a minimum of 1,459 study subjects was deemed necessary, as shown in Eq. (3).

3 $$\begin{array}{c}n=exp\left(\frac{-0.508\ +\ 0.259\ ln\left(\varphi\right)\ +\ 0.504\;ln\left(p\right)\ -\ ln\left(MAPE\right)}{0.544}\right)\end{array}$$

Minimizing overfitting is critical for the model’s predictive accuracy. Riley et al. [34, 35] recommend careful consideration of sample size and the number of predictor variables, particularly with smaller shrinkage rates (≤ 0.1, with an expected shrinkage factor S ≥ 0.9). To ensure an expected contraction rate of 10% and reduce model overfitting, the expected contraction rate R^2^_CS_ was set to 0.1, the expected contraction factor S was set to 0.9, and the number of study variables P was roughly 20. It was calculated that a minimum of 1125 study subjects were needed. As shown in Eq. (4).

4 $$\begin{array}{c}n=\frac p{\left(s-1\right)\;ln\;\left(1-\frac{R_{CS}^2}S\right)}\end{array}$$

Furthermore, the prediction model’s sample size should ensure minimal discrepancy between the developed model and the optimal adjustment value of R^2^_CS_. With maxR^2^_CS_ set at 0.75, the required sample size was calculated to be 497, as detailed in Eqs. (5) and (6).

5 $$\begin{array}{c}S'=\frac{R_{CS}^2}{R_{CS}^2+\delta max\left(R_{CS}^2\right)}\end{array}$$

6 $$\begin{array}{c}n=\frac P{\left(S'-1\right)ln\left(1-\frac{R_{CS}^2}{S'}\right)}\end{array}$$

Therefore, the study necessitated a minimum of 1,459 participants. With a total of 5,469 participants, the sample size was well-suited for the research objectives.

### Model construction and evaluation

Three predictive models—XG Boost, Support Vector Machines, and Random Forests—were developed using Python 3.8.10. The sample data were randomly divided in a 7:2:1 ratio into training, test, and validation sets using the pandas and NumPy libraries in Python.

A comprehensive assessment and comparison of these models were conducted using various metrics, including (1) accuracy, (2) precision, (3) recall, (4) AUC, (5) calibration curve, (6) Brier score, (7) log loss, and (8) calibration-in-the-large, which are defined as follows:

$$Accuracy=\frac{TN+TP}{TN+FP+FN+TP}$$

(1)

$$Precision=\frac{TP}{FP+TP}$$

(2)

$$Recall=\frac{TP}{FN+TP}$$

(3) 

Here, TP (True Positives) refers to correctly classified positive samples, FP (False Positives) to negative samples misclassified as positive, TN (True Negatives) to correctly classified negative samples, and FN (False Negatives) to positive samples misclassified as negative.

(4) AUC: The area under the ROC curve, or AUC, reflects the diagnostic value of the model. An AUC closer to 1 signifies superior diagnostic performance.
(5) Calibration curve: The model’s calibration is more accurate the closer this curve is to the diagonal line.
(6) Brier score: This metric quantifies the model’s calibration degree, with values ranging from 0 to 0.25. Values closer to 0 indicate better calibration; a score of 0.25 suggests the model lacks predictive capability.
(7) Log loss: Commonly used in logistic regression and neural networks, as well as certain variants of the expectation-maximization algorithm, this metric evaluates the probabilistic output of a classifier.
(8) Calibration-in-the-large: This refers to the calibration curve’s intercept. A value closer to 0 indicates more accurate model calibration.

### Statistical analysis

The original database was compiled using Excel 2016. Statistical analyses were conducted with IBM SPSS 24.0. The count data were displayed as composition ratios or rates, and the Chi-square test was used to compare groups of data; ordinal data were similarly described and compared using the Kruskal-Wallis test. COX proportional hazards regression modeling was used to carry out multifactor analysis. With a significance level α set at 0.05, every test was conducted in two-sided.

### Quality control

Investigators strictly followed inclusion and exclusion criteria and were trained uniformly. To guarantee data authenticity, data entry was double-checked, and computer and human verification as well as logical error checks were used. Devoted staff members maintained and calibrated measurement instruments on a regular basis. The data was analyzed using appropriate statistical techniques, guaranteeing the validity of the test results.

### Research findings

During the follow-up period, the incidence of new obesity cases among the study participants varied annually: 1,055 cases in 2019, 120 in 2020, 72 in 2021, and 74 in 2022. By the end of the follow-up, the total number of new obesity cases reached 1,319, comprising 1,246 males and 73 females. The overall obesity prevalence among steelworkers was 24.1%.

### Single-factor analysis

The demographic characteristics of the study population indicated a decreasing risk of obesity with increasing age. Incidence rates were higher among males than females and varied significantly across marital statuses and educational levels (*P* < 0.05) (Table 3).

**Table 3:** Analysis of demographic characteristics of research objects

Variable	Number of cases	incidence (morbidity %)	X^2^/H(K)	*P*
Age (years)			21.007^a^	<0.001
≤ 30	436	158(36.2)		
30~	1436	482(33.6)		
40~	2045	451(22.1)		
≥ 50	1552	228(14.7)		
Nation			4.243	0.039
Han Chinese	5335	1278(24.0)		
Other	134	41(30.6)		
Sex			30.725	<0.001
Male	4965	1246(25.1)		
Female	504	73(14.5)		
Marital Status			10.299	0.036
Unmarried	251	74(29.5)		
Married or remarried	5056	1199(23.7)		
Divorced or widowed	162	46(28.4)		
Education level			34.140^a^	<0.001
Elementary school and below	60	12(20.0)		
Junior high school, high school, or junior college	4077	928(22.8)		
College and above	1332	379(28.5)		
Monthly per capita household income (yuan)			0.840^a^	0.657
<1500	1558	380(24.4)		
1500~	2048	494(24.1)		
≥ 2500	1337	335(25.1)		

^a^Ordinal number information was tested using the K-W test

Behavioral lifestyle analysis of the steelworkers revealed that those with lower DASH diet scores had a significantly higher obesity incidence compared to those with higher scores. Additionally, obesity prevalence was higher among workers who smoked and consumed alcohol. Workers with low physical activity levels also showed a higher incidence of obesity compared to their more active counterparts, underscoring the potential role of unhealthy lifestyles as a risk factor for obesity. These findings are presented in Table 4.

**Table 4:** Analysis of the behavior and lifestyle of the study subjects

Variable	Number of cases	incidence (morbidity %)	X^2^/H(K)	*P*
DASH			33.813^a^	<0.001
<25	2407	679(28.2)		
≥ 25	3062	640(20.9)		
AIS			0.487^a^	0.485
≤ 6	3113	764(24.5)		
>6	1580	369(23.4)		
Smoking			42.111	<0.001
Never smoked	2422	581(24.0)		
Former smoker	1006	63(6.3)		
Current smoker	2041	675(33.1)		
Drinking			34.802	<0.001
Never drinking	3276	734(22.4)		
Former drinker	111	21(18.9)		
Current drinker	2082	564(27.1)		
Physical Activity			21.087^a^	<0.001
Low	201	78(38.8)		
Medium	464	144(31.0)		
High	4804	1097(22.8)		

^a^Ordinal number information was tested using the K-W test

Analysis of occupational hazards indicated an upward trend in obesity prevalence among steelworkers with increasing age. Factors such as shift work, exposure to high temperatures, CO, and occupational stress were identified as obesity risk factors (Table 5).

**Table 5:** Analysis of occupational factor exposure of research subjects

Variable	Number of cases	incidence (morbidity %)	X^2^/H(K)	*P*
Working age (years)			42.599^a^	<0.001
<20	1895	644(3.4)		
20~	1995	430(21.6)		
≥ 30	1580	246(14.8)		
Shift work			22.285	<0.001
Never work shifts	1025	216(21.1)		
Used to work shifts	1026	240(23.4)		
Now working shifts	3418	863(25.2)		
High-temperature exposure			24.189	<0.001
Yes	2075	563(27.1)		
No	3394	756(22.3)		
Noise exposure			0.685	0.408
Yes	1987	444(22.3)		
No	1618	328(20.3)		
Dust exposure			1.594	0.207
Yes	1566	306(19.5)		
No	2040	466(22.8)		
CO exposure			8.219	0.004
Yes	271	85(31.4)		
No	5198	1234(23.7)		
Occupational Stress			16.020	<0.001
Yes	3442	838(24.3)		
No	2027	481(23.7)		

^a^Ordinal number information was tested using the K-W test

### Multifactor analysis

Multifactor analysis of steelworkers’ data was conducted using the Cox proportional hazards model. The influencing factors for obesity in steelworkers were identified as sex, age, smoking status, alcohol consumption, DASH diet score, physical activity, shift work, and CO exposure (Table 6).

**Table 6:** COX regression analysis of factors affecting obesity among steel workers

Variable	B	SE	Wald	df	*P*	Exp(B)	95%CI	
							Lower	Upper
Sex	-0.313	0.132	5.625	1	0.018	0.732	0.565	0.947
nation	0.093	0.164	0.319	1	0.572	1.097	0.795	1.513
Age (years)								
≤ 30			15.705	3	<0.001			
30~	-0.119	0.104	1.300	1	0.254	0.888	0.724	1.089
40~	-0.314	0.159	3.905	1	0.048	0.731	0.535	0.997
≥ 50	-0.639	0.178	12.841	1	<0.001	0.528	0.372	0.749
Education level								
Elementary school and below			1.260	2	0.533			
Junior high school, high school, or junior college	0.034	0.305	0.012	1	0.912	1.034	0.569	1.881
College and above	0.113	0.311	0.132	1	0.716	1.120	0.608	2.061
Marital Status								
Unmarried			4.361	4	0.359			
Married or remarried	0.100	0.139	0.525	1	0.469	1.106	0.843	1.450
Divorced or widowed	0.355	0.210	2.854	1	0.091	1.427	0.945	2.155
Monthly per capita household income (yuan)								
<1500			0.116	2	0.944			
1500~	0.007	0.069	0.009	1	0.923	1.007	0.879	1.153
≥ 2500	0.026	0.078	0.109	1	0.741	1.026	0.881	1.194
Occupational Stress	0.101	0.060	2.774	1	0.096	1.106	0.982	1.245
Sleep quality	-0.056	0.067	0.714	1	0.398	0.945	0.830	1.077
Smoking	0.421	0.061	47.941	1	<0.001	1.524	1.352	1.716
Drinking	0.175	0.063	7.857	1	0.005	1.192	1.054	1.347
DASH	-0.130	0.060	4.765	1	0.029	0.878	0.781	0.987
Physical Activity								
Low			14.326	2	<0.001			
Medium	-0.246	0.154	2.564	1	0.109	0.782	0.579	1.057
High	-0.436	0.128	11.525	1	<0.001	0.646	0.503	0.832
Working age (years)								
<20			5.072	2	0.079			
20~	-0.158	0.130	1.489	1	0.222	0.854	0.662	1.101
≥ 30	-0.335	0.155	4.668	1	0.031	0.715	0.528	0.969
Shift work								
Never work shifts			34.000	2	<0.001			
Used to work shifts	0.106	0.111	0.917	1	0.338	1.112	0.895	1.382
Now working shifts	0.447	0.089	25.309	1	<0.001	1.564	1.314	1.861
High-temperature exposure	0.327	0.074	19.447	1	<0.001	1.387	1.199	1.604
Noise exposure	0.145	0.068	4.553	1	0.063	0.865	0.757	0.988
Dust exposure	0.031	0.074	0.178	1	0.674	1.032	0.892	1.194
CO exposure	1.219	0.236	26.724	1	<0.001	3.384	2.132	5.373

### Model effectiveness evaluation

Incorporating results from both univariate and multivariate analyses, as well as relevant literature, the study selected 10 significant independent variables for the model: age, sex, smoking status, drinking status, DASH diet score, physical activity level, shift work, high-temperature exposure, CO exposure, and occupational stress.

Training on 3828 samples (70%) demonstrated that for the random forest model, precision, AUC, log loss, and calibration-in-the-large were 0.823, 0.873, 0.340, and 0.049, respectively. For the support vector machine model, accuracy, recall, and Brier scores were 0.861, 0.602, and 0.105, respectively. Initially, these two models performed better, with the XG Boost model lagging. Model parameters were refined during training and tested using validation set data. Results from 547 validation samples (10%) showed that the random forest model’s metrics—precision, AUC, Brier score, log loss, and calibration-in-the-large—were 0.684, 0.849, 0.122, 0.388, and 0.051, respectively, surpassing the other models. Testing on 1,094 test set samples (20%) confirmed that the random forest model’s accuracy, precision, AUC, log loss, Brier score, and calibration-in-the-large outperformed the other two models, indicating its optimal overall performance (Table 7).

**Table 7:** Evaluation of three risk models

Evaluation Indicator	Training set			Validation set			Test set		
	XG Boost	SVM	Random forest	XG Boost	SVM	Random forest	XG Boost	SVM	Random forest
Accuracy	0.791	0.861	0.853	0.790	0.829	0.820	0.819	0.868	0.872
Precision	0.633	0.790	0.823	0.577	0.676	0.684	0.571	0.696	0.765
Recall	0.392	0.602	0.524	0.340	0.502	0.411	0.333	0.592	0.481
AUC	0.789	0.851	0.873	0.763	0.826	0.849	0.798	0.908	0.912
Brier score	0.144	0.105	0.107	0.147	0.127	0.122	0.128	0.105	0.104
Log loss	0.448	0.345	0.340	0.459	0.402	0.388	0.409	0.349	0.345
Calibration-in-the-large	0.056	0.052	0.049	0.056	0.056	0.051	0.058	0.054	0.051

**Fig. 2:**
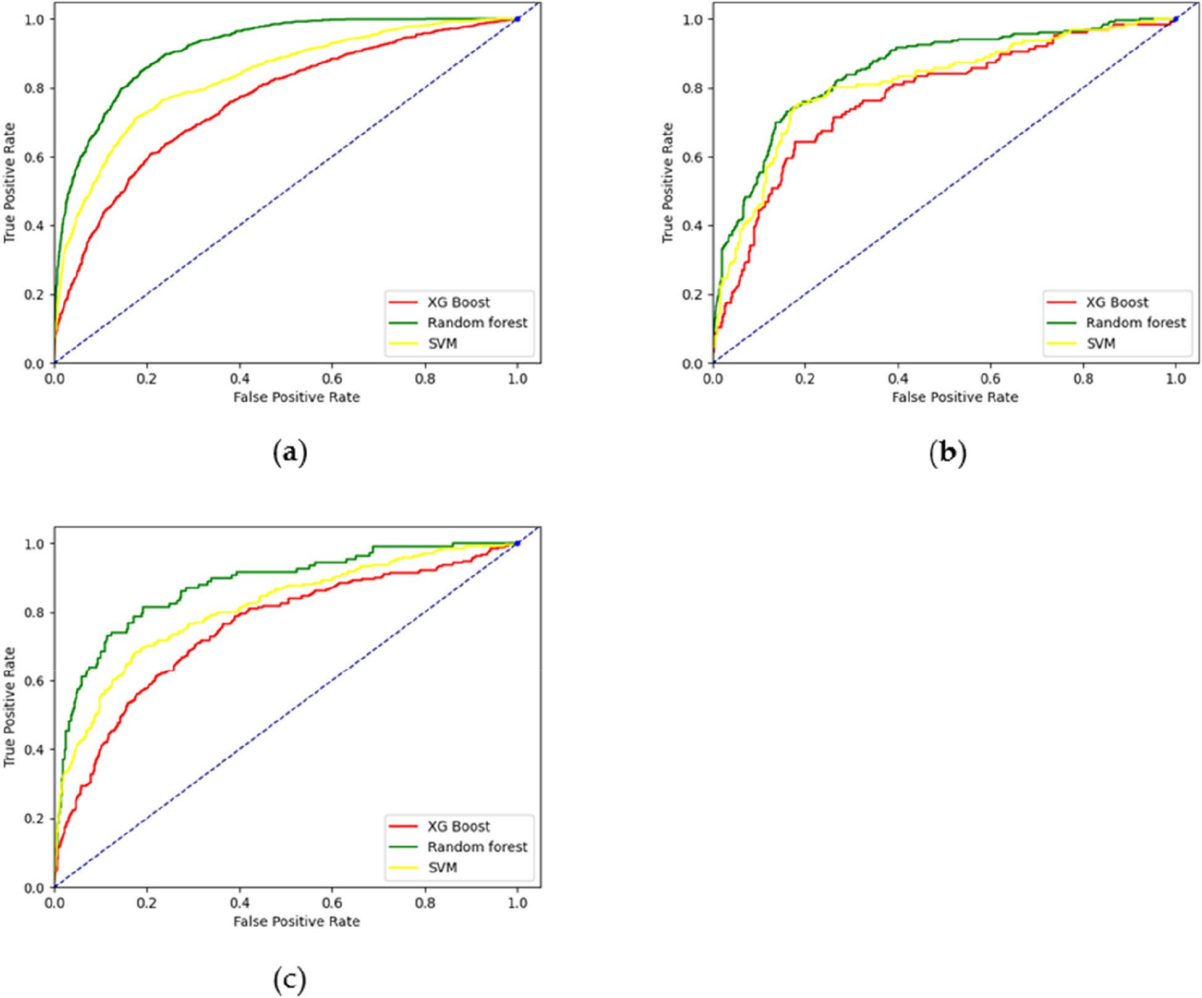
Three models’ ROC curves: **a** Training set; **b** Validation set; **c** Test set

The three models were compared in terms of the Area Under the ROC Curve (AUC). The XG Boost model fared the lowest in the training set, whereas the random forest model surpassed each of the other two. Similar conclusions were noted for the test and validation sets, demonstrating the Random Forest model’s superior predictive capability. These results are illustrated in Fig. 2a-c.

**Fig. 3:**
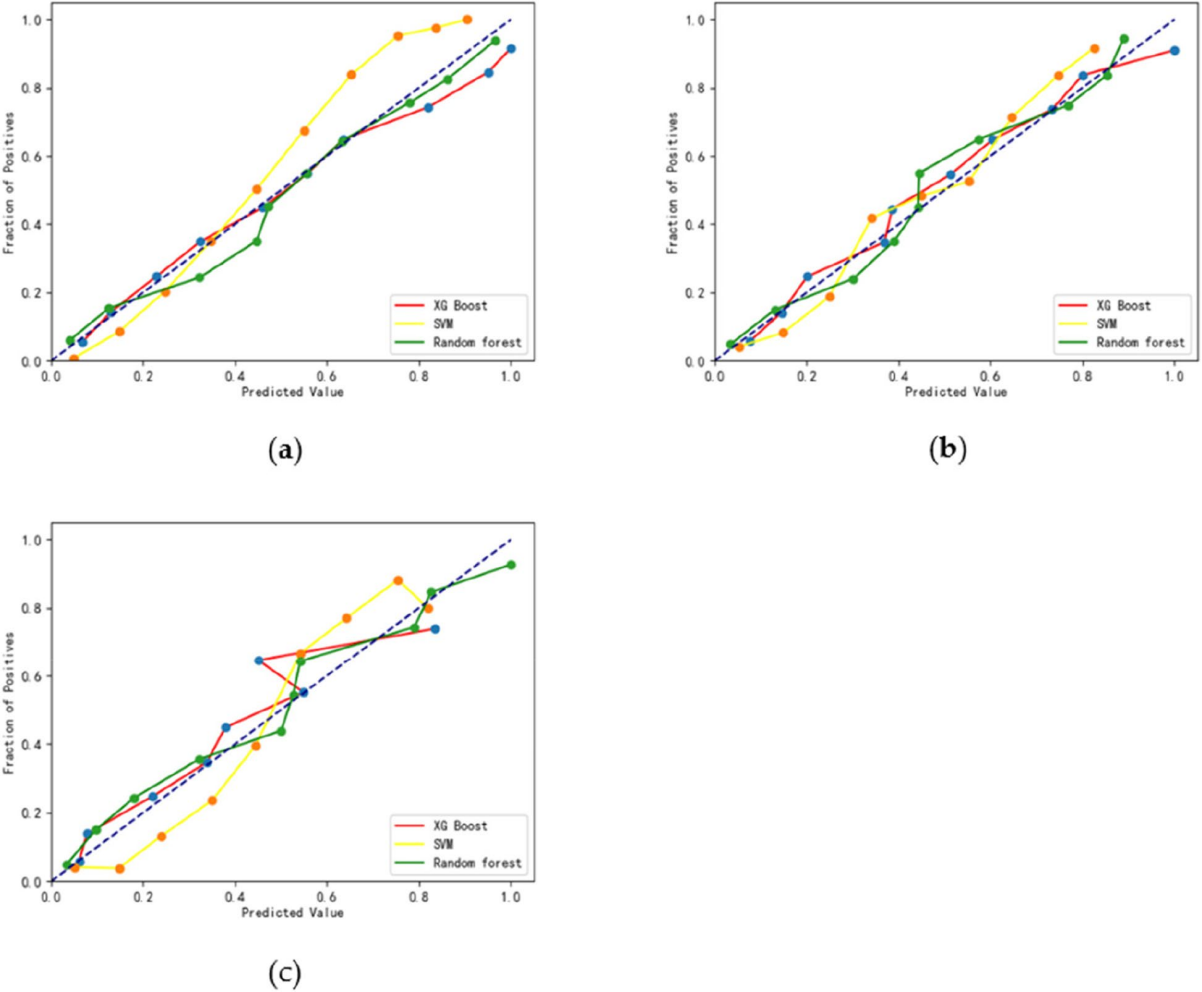
Three models’ calibration curves: **a** Training set; **b** Validation set; **c** Test set

The calibration curves of the random forest model in the training, test, and validation sets were closely aligned with the diagonal, indicating minimal bias. The calibration curves for all three models in the respective sets are displayed in Fig. 3a-c.

**Fig. 4:**
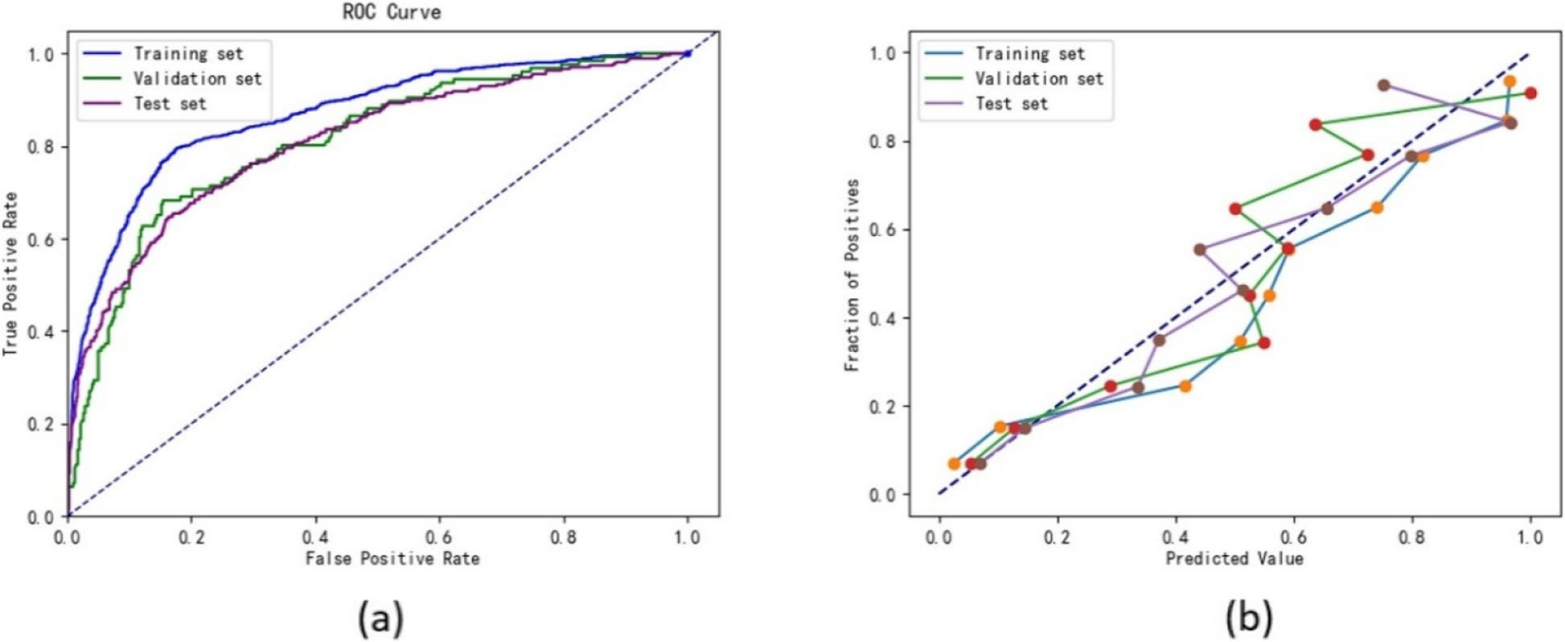
**a** ROC curves of logistic regression; **b** Calibration curves of logistic regression

Additionally, the data were analyzed using a more traditional logistic regression model. This analysis revealed that the logistic regression model’s predictive performance was superior to that of the XG Boost model, yet inferior to the Random Forest and SVM models (Table 8). The calibration and ROC curves for the logistic regression model are presented in Fig. 4a-b.

**Table 8:** Evaluation indicators of logistic regression

Evaluation Indicator	logistic regression		
	Training set	Validation set	Test set
Accuracy	0.838	0.812	0.817
Precision	0.768	0.646	0.720
Recall	0.465	0.405	0.439
AUC	0.867	0.810	0.808
Brier score	0.118	0.135	0.136
Log loss	0.383	0.425	0.433
Calibration-in-the-large	0.070	0.069	0.069

## Discussion

Timely identification, diagnosis, and treatment are of great help for tertiary prevention. Machine learning techniques have recently enhanced the field of disease risk prediction. While obesity prediction in the general population has been extensively studied, research on occupational populations, particularly steelworkers, is limited. Occupational hazards are recognized risk factors for obesity in this group, but studies focusing on steelworkers are scarce [16, 36–38]. Steelworkers’ lifestyles, heavily influenced by their work environment and conditions, underscore the need to identify modifiable obesity risks in this demographic to develop effective prevention methods and policies. This study, conducted over five years with 5469 iron and steel workers, found a five-year cumulative obesity prevalence of 24.1% among these workers. The study suggests that obesity in steelworkers is influenced not only by lifestyle factors but also by various occupational factors. By constructing and comparing Random Forest, XG Boost, and Support Vector Machine risk prediction models, and referencing classical logistic regression model metrics, the Random Forest model emerged as the most effective in this study.

In this research, factors such as age, gender, DASH diet score, drinking and smoking habits, degree of physical activity, shift work, high-temperature exposure, CO exposure, and occupational stress were identified as significant in the development of obesity among steelworkers. Notably, shift work, high-temperature exposure, CO exposure, and occupational stress are distinct factors for this group compared to the general population. The obesity rate was notably higher among workers engaged in or with a history of shift work, possibly due to disruptions in circadian rhythms and sleep-wake cycles, leading to abnormal lipid metabolism and insulin secretion disturbances. This finding aligns with Grundy et al.‘s study [15]. Moreover, shift work often coincides with night light exposure, another significant factor in obesity development [39]. The effects of high-temperature exposure on obesity are not widely researched. Prolonged high-temperature exposure may reduce brown adipose tissue activity, necessary for maintaining constant body temperature, thus decreasing energy expenditure and increasing susceptibility to insulin resistance and fat accumulation. Epidemiological studies indicate a negative correlation between brown adipose tissue and obesity prevalence, with individuals having higher proportions of this tissue at a lower obesity risk [40, 41]. The findings on CO exposure in this study were unexpected. Prolonged excessive CO exposure may inhibit heme oxygenase (HO), leading to disturbances in lipid metabolism and thereby contributing to obesity development. In both animal and human studies, upregulation of HO has been shown to ameliorate obesity and enhance vascular function [42, 43]. CO, being a toxic and hazardous gas, necessitates vigilance in industrial settings. Effective measures are required to protect workers from CO exposure, including enhanced protective measures and improved ventilation, especially considering CO generation is often an inevitable aspect of production operations. Mental health disorders and negative emotions stemming from occupational stress can contribute to abnormal eating behaviors and sedentary lifestyles, further escalating the risk of obesity [44]. Associated depression and anxiety frequently lead to insomnia, a significant obesity risk factor [45, 46]. In this study, lifestyle factors that contribute to obesity, such as smoking and alcohol consumption, were found, and the conclusion is consistent with previous studies. Wannamethee SG et al. [10] discovered that heavy drinkers typically had higher BMIs than nondrinkers or moderate drinkers, but could reach similar BMI levels to nondrinkers after five years of controlled drinking. A 2017 study in Korea by Rha EY et al. [47] reported a positive association between alcohol consumption and central obesity prevalence. Furthermore, epidemiological evidence indicated a positive correlation between obesity prevalence and smoking duration, a finding echoed in a related study in China [48]. In a cross-sectional analysis of a Chinese multiethnic cohort, Tang Dan et al. concluded that adherence to the DASH diet reduces obesity risk [49]. The outcomes of this study support earlier findings by demonstrating that steelworkers who score higher on the DASH diet had a lower risk of obesity.

This study not only evaluated several models mentioned in the text but also compared logistic regression, a traditional statistical prediction model, with the three aforementioned machine learning models. The limitations of logistic regression, particularly when dealing with non-independent disease risk factors and potential nonlinear relationships, impacted its predictive accuracy. Adjustments to the logistic regression model, such as transforming numerical variables into ordered categorical variables, improved its performance. This aligns with previous findings where logistic regression’s predictive power diminishes if data requirements are not met [50]. Casanova et al. [51] compared Random Forest and logistic regression in classifying 3,443 patients with diabetic retinopathy and found Random Forest to be more accurate. XG Boost, an enhancement of the GBDT-based Boosting algorithm [52]. Despite its effectiveness, XG Boost was not the preferred method for predicting obesity in steelworkers due to its relatively lower performance on evaluation indices compared to the other models. Support Vector Machines have shown promise in previous obesity studies [53], and in this study, while only the recall in the final test set was higher than that of the Random Forest model, the differences in other indicators were minimal. However, this model requires data preprocessing and parameterization for large sample sizes and presents challenges in monitoring and visualization. The Random Forest model has excelled in chronic disease prediction. Alghamdi et al. [54] used methods including decision trees, naive Bayesian, logistic regression, and random forest for diabetes prediction in the Henry Ford Exercise Trial project database, finding Random Forest to be the most effective. In this study, the Random Forest model not only effectively differentiated between normal and abnormal BMI but also showed the highest agreement between predicted and actual results, making it particularly suited for analyzing obesity data among steelworkers. Additionally, the model can attribute internal importance to predictor variables, aiding in subsequent model visualization. Based on these findings, the Random Forest model is recommended for obesity risk prediction in steelworkers.

### Study strengths and limitations

This five-year follow-up study included 5,469 individuals and was based on the Beijing-Tianjin-Hebei cohort. Its findings are highly complete and credible. Unlike previous obesity studies, this research incorporated both conventional and occupational factors, aligning the conclusions more closely with the characteristics of the occupational population. This study is novel in using machine learning methods to predict obesity risk in steelworkers, providing new methodological support for future obesity-related disease prevention. Although previous studies have shown associations between high temperature and CO exposures with obesity, their specific impacts on obesity development in steelworkers were not explored until now.

However, the study has limitations. It did not include genetic data from steelworkers, considering genetics are immutable and their inclusion would not aid in providing practical obesity prevention recommendations. Furthermore, this study only built and completed internal validation of the model for predicting the risk of obesity in steelworkers; external validation was not conducted. Moreover, while the optimal model for predicting obesity in steelworkers was identified, further investigation is needed on how to effectively visualize and apply this model.

## Conclusion

A five-year observational study involving 5,469 steelworkers found that age, sex, drinking and smoking habits, DASH diet score, physical activity level, shift work, exposure to high temperatures, and CO exposure were the main factors influencing the development of obesity in this group. A Random Forest Model specifically suited for predicting obesity in steelworkers was successfully developed and demonstrated superior predictive ability compared to other models.

## Data Availability

The datasets used in this study are available from the corresponding author upon reasonable request.

## Abbreviations

SVM: Support vector machine
RF: Random forest
ML: Machine learning
BMI: Body mass index
AIS: Athens insomnia scale
DASH: Dietary Approaches to Stop Hypertension
AUC: Area under the ROC curve
